# Computed tomography manifestations and pathological features of intra-abdominal desmoplastic small round cell tumor

**DOI:** 10.12669/pjms.41.3.10154

**Published:** 2025-03

**Authors:** Ashan Pan, Xunze Shen, Qiande Qiu

**Affiliations:** 1 Ashan Pan Department of Radiology, Yueqing People’s Hospital, Wenzhou 325600, China; 2 Xunze Shen Department of Radiology, Shaoxing People’s Hospital, Shaoxing 312000, China; 3 Q**i**ande Qiu Department of Imaging, Wenzhou People’s Hospital, Wenzhou 325000, China

**Keywords:** Computed tomography, DSRCT, Intra-abdominal, Manifestations

## Abstract

**Objective:**

To explore the computed tomography (CT) manifestations and pathological features of intra-abdominal desmoplastic small round cell tumor (DSRCT).

**Methods:**

Retrospective analysis was conducted on the CT manifestations and pathological features of 11 patients with intra-abdominal DSRCT in Yueqing People’s Hospital, Shaoxing People’s Hospital and Wenzhou People’s Hospital from January 2010 to June 2022.

**Results:**

Among 11 patients, four cases were with solitary tumor and seven cases were with multiple tumors. There were five cases with elliptical shape, smooth edges and clear boundary and six cases with irregular shape and unclear boundary. The CT plain scan showed the uneven density in tumor. There were six cases with necrosis and cystic transformation in central tumor, and five cases with small patchy or strip-shaped calcification within the tumor. The enhanced CT scan showed uneven enhancement. There were seven cases of mild enhancement and four cases of moderate enhancement. Five cases showed strip or grid-like enhancement within tumor, and five cases showed increased and thickened vascular shadows around the tumor. The immunohistochemistry showed nine cases were positive for AE1/AE3, CK, Vim and Desmin, eight cases were positive for EMA, seven cases were positive for NSE, and three cases were positive for CGA, SYN, CD56 and CD99.

**Conclusion:**

In CT manifestations, the intra-abdominal DSRCT present the solitary or multiple tumors, with uneven density. There are often cystic changes and small patchy calcification within the tumor, with mild to moderate uneven enhancement.

## INTRODUCTION

Desmoplastic small round cell tumor (DSRCT) is a rare and highly invasive malignant tumor. It is commonly found in the abdominal and pelvic cavities.^1^,^2^ In the diagnosis, DSRCT needs to be differentiated from non-organ derived tumors, mainly including neuroblastoma, rhabdomyosarcoma, primitive neuroectodermal tumor, malignant mesothelioma, and undifferentiated pleomorphic sarcoma.^3^ The computed tomography (CT) is an important method to diagnose the lesions in the body.^4^ At present, there is a lack of sufficient understanding about CT manifestations of DSRCT, which can easily lead to the misdiagnosis. The reports on the CT manifestations of DSRCT are rare.^5^,^6^ In view of this, this study conducted a retrospective analysis on the characteristics of CT findings in 11 patients with intra-abdominal DSRCT confirmed by pathology, in order to improve the understanding and diagnostic accuracy of DSRCT.

## METHODS

This study collected the data of 11 patients with intra-abdominal DSRCT confirmed by pathology who were admitted to Yueqing People’s Hospital (Wenzhou, China), Shaoxing People’s Hospital (Shaoxing, China) and Wenzhou People’s Hospital (Wenzhou, China) from January 2010 to June 2022. Among these 11 cases, there were five males and six females. The age range was 24-64 years, with an average of 39.9±12.8 years. All 11 patients presented the abdominal pain or bloating, with seven cases complicated with had vomiting, seven cases complicated with ascites, six cases complicated with weight loss, five cases complicated with bowel habit change, and three cases complicated with lower limb edema. Among 11 patients, the DSRCT was confirmed by surgical pathology in eight cases and biopsy pathology in three cases.

### Ethical Approval:

This study was approved by the ethics committee of Yueqing People’s Hospital (No. YQYY202400135; Date: January 15, 2024). Written informed consent was obtained from all participants.

### CT scan:

Light Speed Pro16 layer MSCT scanner (GE HealthCare, USA) and Somatom Emotion 16 layer MSCT scanner (Siemens, Germany) were adopted for the CT scan. The scan conditions were as follows: voltage, 120 kV; current, 250 mA; field of view, 250 mm × 250 mm; layer thickness, 1.5 mm; interval, 1.5 mm; matrix, 512 × 512. In the contrast-enhanced CT scan, 100 ml of 300 mg/ml iohexol was injected into the elbow vein, with dose of 1.5 ml/kg and flow rate of 3.0 ml/s. The images of arterial phase, portal venous phase and delayed phase were collected at 30 s, 60 s and 120 s after injection, respectively. Both plain and enhanced CT scans were performed in all 11 patients.

### Pathological diagnosis:

Specimens of patients were fixed in 4% formaldehyde solution, and embedded in paraffin. The hematoxylin-eosin staining was performed. In addition, the immunohistochemical staining was conducted using the streptomycin antibiotin peroxidase method. The antibodies such as AE1/AE3, Vim, Desmin, EMA, NSE, CGA, SYN, CD56, CD99, AyoD1, LCA and others were purchased from Dako Inc. (Copenhagen, Denmark).

## RESULTS

### Location, size, shape and boundary of tumor:

In 11 patients of with intra-abdominal DSRCT, four cases were with solitary tumor, including two cases located in upper left abdomen, one case located in lower abdomen, and one case located in pelvic cavity. Seven cases were with multiple tumors, including one case in left upper abdomen and right lower abdomen, one case in right middle lower abdomen and peri-liver, one case in left middle abdomen and pelvic cavity, one case in right lower abdomen and pelvic cavity, one case in both sides of upper abdomen and peri liver, one case in posterior part of bladder and ileum mesentery, and one case in lower abdomen and pelvic cavity. In four cases of solitary tumor, the maximum diameter was 9.6-20.5 cm, with an average of 12.5±3.3 cm. In seven cases of multiple tumors (total 16 tumors), the maximum diameter was 2.5-16.0 cm, with an average of 5.5±3.8 cm. In five cases, the tumor had elliptical shape, smooth edges and clear boundary. In six cases, the tumor had invasive growth, with irregular shape and unclear boundary with surrounding tissues.

### Plain and enhanced CT manifestations:

**Fig.1:**
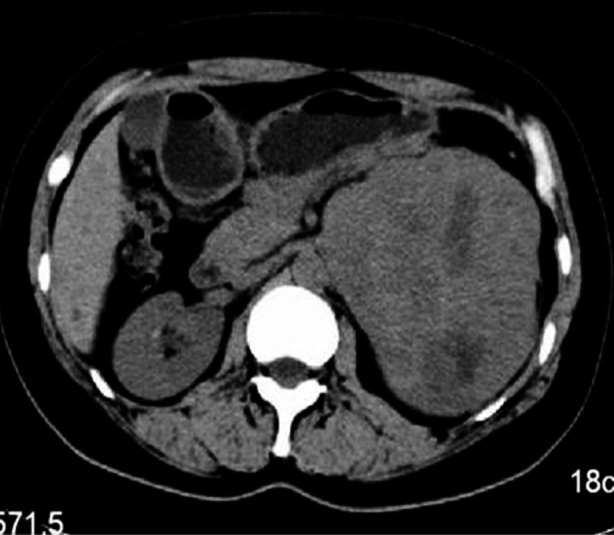
Plain CT manifestation of intra-abdominal DSRC. The tumor with 11.0 × 12.5 cm was in upper left abdomen, with clear boundary, uneven density, and patchy necrotic and cystic area within the tumor; the CT value was 36 HU.

Plain CT scan showed that, the density of tumor in 11 patients was uneven. The CT value was 29-51 HU, with an average of 38.9±6.6 HU. The parenchymal part of tumor presented hypodense in five cases, isodense in five cases and slight hyperdense in one case. There were six cases with necrosis and cystic transformation in central tumor, and five cases with small patchy or strip-shaped calcification within the tumor (Fig.1).

**Fig.2:**
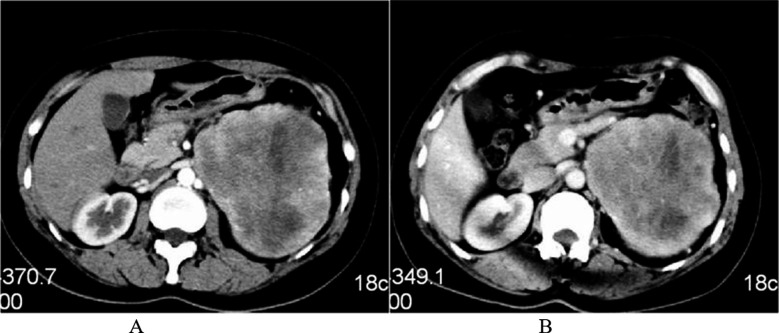
Enhanced CT manifestations of intra-abdominal DSRC. (A) There was moderate uneven enhancement in arterial phase, with no enhancement in necrotic and cystic areas; the CT value was 57 HU. (B) There was continuous mild uneven enhancement in portal venous phase; the CT was 63 HU. Invasion and metastasis.

Enhanced CT scan showed that, the CT value in 11 patients was 41-85 HU in arterial phase (average, 57.0±10.6 HU) and 47-83 HU in portal venous phase (average, 62.5±9.0 HU). All tumors showed uneven enhancement, with no enhancement in the necrotic and cystic area. There were seven cases of mild enhancement and four cases of moderate enhancement. Five cases showed strip or grid-like enhancement within the tumor, and five cases showed increased and thickened vascular shadows around the tumor (Fig.2).

Among 11 patients, six cases showed tumor invasion of adjacent tissues and organs. In two cases the tumor invaded the adjacent intestinal ducts, causing thickening of intestinal wall and formation of soft tissue masses. In two cases the tumor invaded the middle segment of ipsilateral ureter, causing ureteral dilation and hydronephrosis. In other two cases the tumor invaded the left kidney, spleen and pancreatic tail. As for the metastasis, there were seven cases of retroperitoneal lymph node metastasis in abdominal cavity, three cases of liver or perihepatic metastasis, two cases of rib, femur and sacrum metastasis, and one case of lung metastasis.

### Preoperative misdiagnosis:

All 11 cases of tumor were misdiagnosed as other tumors by preoperative CT. Two cases originated from the retroperitoneum and invaded the left lumbar muscle, with unclear boundary, and they were misdiagnosed as undifferentiated pleomorphic sarcoma. Two cases located on the left side of pelvic cavity, and were misdiagnosed as metastatic tumors (the patient had cervical cancer surgery three years ago). Two cases located behind the peritoneum and invaded the surrounding tissues, with unclear tissue origin, and they were diagnosed as unknown malignant tumor. Two cases located in front of left kidney, and invaded the left kidney, spleen and tail of pancreas, with unclear boundary to kidney tissue, and they were misdiagnosed as malignant kidney tumor. One case originated from the small intestine mesentery, and invaded the pancreatic head periphery and duodenum, and it was misdiagnosed as malignant stromal tumor of duodenum. One case originated from mesenteric root of jejunum, with unclear boundary to jejunum, and it was misdiagnosed as jejunal stromal tumor. One case was the multiple tumors, with multiple enlarged retroperitoneal lymph nodes, and it was misdiagnosed as lymphoma.

### Surgery finding and pathology:

All 11 cases of DSRCT were confirmed by pathology from surgery (eight cases) or biopsy (three cases). Five cases showed clear tumor boundary during surgery and underwent the complete resection. Six cases showed tumor invasive growth, with lobulated and irregular shape. Among them, three cases were closely adhered to surrounding blood vessels and underwent the partial tumor resection. Other three cases had extensive metastasis to peritoneum, omentum, mesentery and surrounding intestines, and only the biopsy was taken. The cut surface of tumor appeared gray white or gray yellow, with tough texture and visible necrotic lesions of varying sizes.

**Fig.3:**
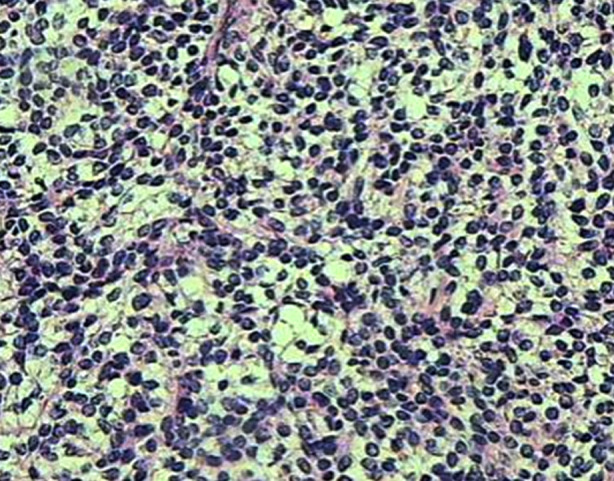
Microscopic presentation of DSRCT. The tumor cells were in nest-like or clustered structure, with characteristic fibrous interstitial bands (hematoxylin-eosin, × 400).

Under the microscope, the tumor cells are small, mostly round or oval in shape, and tightly arranged. The cells aggregated into a nest-like or clustered structure, with clear boundary, varying size, and different shape. The stromal cells outside tumor cell nest were buried in the dense collagen matrix, including fibroblasts, myofibroblasts, and intermediate cells (Fig.3).

**Fig.4:**
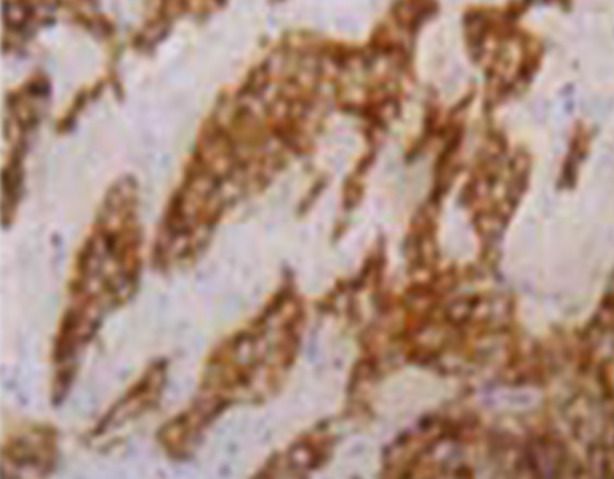
Immunohistochemical presentation of DSRCT. The tumor cells showed diffuse strong positive expression of AE1/AE3 (streptomycin antibiotin peroxidase, × 400).

Immunohistochemical examination showed nine cases were positive for AE1/AE3 (Fig.4), CK, Vim and Desmin, eight cases were positive for EMA, seven cases were positive for NSE, and three cases were positive for CGA, SYN, CD56 and CD99. Both LCA and YnyoD1 were negative in all cases.

Follow up showed that, 11 patients postoperatively survived for 3-4 months, with an average of 11.5±9.4 months. Among them, two cases survived for three months, two cases survived for four months, two cases survived for five months, two cases survived for seven months, one case survived for nine months, one case survived for 16 months, and one case survived for 42 months.

## DISCUSSION

This study explored the CT manifestations and pathological features of intra-abdominal DSRCT. Results indicated that, the CT plain scan showed the uneven density in tumor. There were six cases with necrosis and cystic transformation in central tumor, and five cases with small patchy or strip-shaped calcification within the tumor. The enhanced CT scan showed uneven enhancement. There were seven cases of mild enhancement and four cases of moderate enhancement. Five cases showed strip or grid-like enhancement within tumor, and five cases showed increased and thickened vascular shadows around the tumor. The immunohistochemistry showed nine cases were positive for AE1/AE3, CK, Vim and Desmin, eight cases were positive for EMA, seven cases were positive for NSE, and three cases were positive for CGA, SYN, CD56 and CD99.

DSRCT is a rare malignant tumor that is more common in children and adolescents, with a male to female ratio of 4:1.^7^ It often occurs in the abdomen and pelvis, and can also be seen in various parts of the body.^8^ The clinical manifestations of DSRCT are mainly the abdominal distension and pain, palpable masses, and even ascites sign.^9^ DSRCT is a highly malignant tumor. Most patients have peritoneal, hepatic, and distant metastasis at the initial diagnosis, making the surgical removal of lesion difficult.^10^,^11^ Our study analyzed the data of 11 patients with intra-abdominal DSRCT. Among these 11 patients, only five cases received the complete tumor resection, and three patients received the partial tumor resection. Other three cases had extensive metastasis to peritoneum, omentum, mesentery and surrounding intestines, so only the biopsy was taken. DSRCT has a high malignancy and extremely poor prognosis. Despite the use of comprehensive methods such as radiotherapy, chemotherapy and surgery, the satisfactory therapeutic effects cannot be achieved.^12^ Nakayama et al.^13^ have summarized the cases reported in recent literature and found that the survival time of DSRCT patients was only four to 42 months. In our study, 11 cases postoperatively survived for 3-4 months, with an average of only 11.5±9.4 months.

Previous studies^14^-^17^ have shown that, in plain CT scan, DSRCT presents the soft tissue masses in the abdominal or pelvic cavity, with varying size and unclear boundary. The shape is often quasi circular, elliptical, or irregular. There are often areas of bleeding and necrosis within the tumor. In enhanced CT scan, the tumor reveals the mild to moderate enhancement. Based on the results of our study and previous reported researches, we have experiences for the CT features of DSRCT as follows:

Firstly, the tumor tissue origin of DSRCT is difficult to determine. DSRCT presents the large solitary mass, multiple irregular nodules or multiple masses in abdomen and pelvis, with uneven density. Due to the large size of solitary mass or uneven distribution of multiple nodules, it is difficult to determine the origin of tumor. In our study, seven cases were with multiple masses (a total of 16 masses), of which six cases showed the invasive growth, with irregular shape and unclear boundary with surrounding tissues, making it impossible to determine the tumor origin.

Secondly, DSRCT is prone to invasion of surrounding tissues and metastasis. When the tumor site is closely related to the omentum, mesentery, and peritoneum, the omentum, mesentery and peritoneum implantation as well as adjacent and distant metastasis often occur. The larger tumor often invades the adjacent tissues and organs and forms the lumps or nodules, resulting in unclear boundary to surrounding tissues. When the tumor is adjacent to ureter or intestinal tract, it often invades or compresses the ureter or intestinal tract, leading to secondary hydronephrosis or intestinal obstruction. In addition, the DSRCT patients are often complicated with abdominal, retroperitoneal, and bilateral inguinal lymph node enlargement, with liver or lung metastasis in the late stage. In our study, 54.5% (6/11) patients were with tumor invasion of adjacent tissues and organs, 63.6% (7/11) patients had retroperitoneal lymph node metastasis in abdominal cavity, and 54.5% (6/11) patients had the liver/perihepatic, rib, femur, sacrum or lung metastasis.

Thirdly, the mass density of DSRCT is uneven. When the tumor becomes large, the necrosis and cystic change within tumor, bleeding, or a large amount of fibrous tissue appear, with small patches of calcification within tumor, resulting in uneven density on plain CT scan.^18^ Xiang et al.^19^ have reported that, 77.8% (7/9) of DSRCT patients have the cystic necrosis in the center of tumor. In our study, 54.5% (6/11) DSRCT patients had necrosis and cystic transformation in central tumor, which is similar with above study.

Fourthly, the enhanced CT scan of DSRCT shows the mild to moderate uneven enhancement. The stromal cells of tumor are mainly composed of fibroblasts and myofibroblasts, covering the proliferative collagen tissue, so the majority of patients show mild and continuous enhancement. Due to the large tumor size, the necrosis, cystic change, bleeding and calcification are prone to occur within the tumor, so most cases present the uneven enhancement. In our study, all cases showed mild to moderate uneven enhancement, with 45.5% (5/11) cases showing strip or grid-like enhancement within tumor, and 45.5% (5/11) cases showing increased and thickened vascular shadows around the tumor.

Based on the results and our above experiences, our study has provided an important reference for improving the understanding and diagnostic accuracy of DSRCT, with obvious clinical significance. Due to the rarity of DSRCT, there is overlap in its imaging findings with other tumors or inflammatory lesions. Therefore, more detailed and accurate identification of DSRCT still needs to be further studied.

### Limitations:

The sample size of this study is relatively small. Larger sample size will make the results more convincing. In our next studies, the sample size should be further increased for obtaining more satisfactory findings.

## CONCLUSIONS

The intra-abdominal DSRCT is a highly malignant tumor. In histological characters, the tumor cells present the nest-like or clustered structures, with irregular shape and varying size. They are buried in proliferative fibrous connective tissue. When CT scans show the irregular-shape mass in abdominal and pelvic regions, with mild to moderate uneven enhancement, invasion of surrounding tissues, and proximal and distant metastasis, it may indicate the possibility of DSRCT.

### Authors’ contributions:

**QQ:** Designed this study and prepared this manuscript

**AP:** Literature search, Collected and analyzed clinical data

**XS:** Literature search, Critical review.

All authors have read, approved the final version and are also , and were responsible and accountable for the accuracy or integrity of the work.
